# Doctors on the move: a qualitative study on the driving factors in a group of Egyptian physicians migrating to Germany

**DOI:** 10.1186/s12992-018-0434-x

**Published:** 2019-01-07

**Authors:** Marwa Schumann, Asja Maaz, Harm Peters

**Affiliations:** 1 Dieter Scheffner Center for Medical Education and Educational Research, Dean’s Office of Student Affairs, Charité - Universitätsmedizin Berlin, Free and Humboldt University Berlin, Campus Charité Mitte, Charitéplatz 1, 10117 Berlin, Germany; 2 Medical Education Department, Alexandria Faculty of Medicine, Alexandria University, Alexandria, Egypt

**Keywords:** Medical migration, Egyptian physicians, Germany, Driving forces, Push and pull factors, Facilitators and barriers, Licensing and registration of foreign physicians, Qualitative study, Social networks

## Abstract

**Background:**

Migration of physicians has become a global phenomenon with significant implications for the healthcare delivery systems worldwide. The motivations and factors driving physician’s migration are complex and continuously evolving. Purpose of this study is to explore the driving forces in a group of Egyptian physicians and final-years medical students preparing to migrate to Germany.

**Methods:**

A qualitative study was conducted based on social constructivism epistemology. In five focus group discussions, there participated a total 12 residents and 6 final-year medical students from 7 different training and workplace locations in Egypt. The participants provided information about their motivation and planning for migration. We applied a coding framework based on the concept of push/pull factors and barriers/facilitators for migration, and used Atlas.ti software for analysis.

**Results:**

The thematic analysis indicated that the migration within the study’s participants results from a specific weighting of push and pull factors. Push factors are considered to be more important than pull factors. Factors related to professional development play a leading role. The route of migration towards Germany is mainly determined by the low hurdle registration and licensing requirements in this destination country compared to other countries. In some cases, Germany is regarded as a “transit country”, a step on the road to other European countries. The intent, planning and preparation of migration is assisted considerably by the local formation of a community and culture of migration with multiple ways for information exchange, identity building and social support through face-to-face and online channels.

**Conclusions:**

This study specifies – in a group of Egyptian physicians and final-year medical students – the perceived push and pull factors which influenced their intent to migrate to Germany. In addition to the general wealth gap, their particular route of migration is mainly determined by the requirements in licensing and registration procedures for foreign physicians in the potential destination country. The planning and preparation of a move is substantially facilitated by their joining a social network and a community of migrating physicians.

## Background

Migration of skilled heath care professionals in general, and physicians in particular, has become a global movement phenomenon [1–3]. Migration takes place along the wealth gap, commonly from less-developed to more-developed countries and regions around the world [1, 2, 4–6]. This phenomenon has a significant impact on the quality of healthcare systems in the source countries, the “home countries of the professionals who travel to work abroad” as well as destination countries “that recruit or accept health professionals” [7, 8]. The driving forces behind the how and why migration occurs are complex in nature and continuously evolving. While the physicians’ migration phenomena have several features in common, new facets still continue to emerge and become unrevealed [9, 10]. In this qualitative study, we explore the driving forces in a group of Egyptian physicians who are planning to migrate to Germany.

The routes of migrating physicians can show specific patterns and directions. For instance, physicians from Pakistan move to the UK, UK physicians move to Canada, and Canadians move to the USA [11], thereby building a chain where physicians are continuously moving from one country to another which has a perceived higher living or health care standard. This migration pattern has been named in the literature the “medical carousel phenomenon”, a term which evokes the impression that all stops are equal, which is actually not the case [11, 12]. The World Health Organisation (WHO) issued a Global Code of Practice in 2010 to mitigate the impact of health profession migration on health care delivery; however this has had little effect on migration practices [13].

Several theories have evolved about international migration in general, e.g. the chain migration theory, the network theory, or the institutional theory [14]. With regard to the migration of physicians in particular, the concept of pull and push factors has emerged as a practical framework to explore and study the underlying driving forces in different contexts and around the world [4, 5, 15, 16]. Push factors represent “factors in a health system or country that repel or facilitate the movement of health workers away from that system or country” ([17], p. 45). Pull factors embody “factors in a health system or country that attract or facilitate the movement of health workers towards that system or country” ([17], p. 45). The commonly found pull und push factors have been classified into a) financial factors related to salary structure and healthcare facilities, b) professional factors related to the quality of medical training and working conditions, and c) general sociopolitical factors related to the political climate and general security [4, 5, 17–24].

In addition to push and pull factors, physicians’ migration is shaped by facilitators and barriers to mobility, for instance, visa procedures, regulation and legislation for working as a physician, active recruitment as well as human resource and health policies [15, 17, 25]. The most commonly described “barriers to mobility” are visa procedures and licensing and registration requirement for migrating physicians [10, 26]. In turn – although it is far less investigated – mobility is facilitated through the formation of social networks of migrated physicians and those with the intention to migrate. These networks can provide various forms of support and allow exchange of critical information through various channels, among peers preparing for migration and between physicians already working abroad, as well as those who plan to follow, including positive role-modelling. These social networks allow members to develop their own identity and the establishment of their shared beliefs and practices, reflecting the formation of a community and culture around the theme migration [10, 27].

Egypt represents a lower-middle income country in the Middle East. Since 2011, the Egyptian sociopolitical situation has been shaped by a wave of political instabilities related to the Arab Spring uprising. The population is growing quickly. Approximately half of the 81 million Egyptians are between the ages of 15 and 29 years. The unemployment rate is currently 9.7% [28, 29]. Egypt suffers from a shortage of physicians although an average of 10,000 medical students graduates annually from 24 public and 3 private medical schools. The shortage is attributed mainly to the emigration of both qualified trainers and graduates due to low job satisfaction, and a search for better training opportunities [30, 31]. In 2016, the density of physicians was estimated to be 1 physician per 12,285 inhabitants [32]. The emigration of physicians abroad contributes substantially to the physician shortage in Egypt, a loss that cannot be replaced by recruitment of health care personal from Sudan und Rwanda [3, 33]. Common destination choices for Egyptian physicians include Gulf countries, Australia and the European Union (EU), including Germany [3, 12, 34].

Germany is a high-income industrialized European country with a constantly aging population [35]. While the density of physicians in the country is high (1 per 214 inhabitants), there is at the same time a relative shortage of physicians, especially in rural regions. Over the last decade, Germany has experienced a sharp increase in foreign-trained physicians which makes the migration process worth exploring [2, 36, 37]. Currently, 11% of practicing physicians in Germany are foreign born or trained [38]. Germany is a member state of the EU, in which a legal framework regulates mutual recognition of professional qualification and the free mobility of physicians within the EU member states. However, there is no clear regulation for the licensing and registration for non-EU physicians [15, 39]. In the current situation, getting the recognition of professional qualification in one EU country would automatically make them eligible for recognition in any other EU country. This may pose problems because the standards for licensing and registration of non-EU physicians differ across the EU member states [39, 40], a feature which may likely influence the migration routes of non-EU physicians.

In this qualitative study, we explored the driving forces for migration of physicians in a cohort of Egyptian physicians and final-year medical students preparing to move to Germany. A series on focus groups was conducted in Alexandria, Egypt. The data are analysed using a framework based on push and pull factors as well as on mobility barriers and facilitators.

## Methods

### Study design and setting

Social constructivism epistemology is the underpinning theory for this qualitative study. We explored factors driving immigration of Egyptian physicians to Germany as “being constructed through social interaction” [41].

The study was conducted from February through May 2017 in Alexandria, Egypt. The sampling frame was Egyptian physicians and final-year medical students attending the “German for doctors” course; a 3-week preparatory course for the medical language examination in Germany that takes place in the Goethe Institute and the Medical Syndicate.

With the aim of stimulating interaction among group members, focus groups were heterogeneous as to the status of participants [42]. Unlike the usual number for focus groups (7–10 people), “mini-focus groups” of 5 or less participants were designed for this research to make a compromise between the width and the breadth of data and take into consideration the busy life style of clinical work [43–45]. We employed a maximum variation sampling strategy: different participants (medical students and residents of different specialties) in various sites (university, ministry of health, private and military hospitals) were chosen to allow the study of a broad range of experiences and maximize opportunities to elicit data [41, 46].

Residents were individuals who had already completed their house officer training and who were carrying out their residency within various specialty fields (e.g. radiology, cardiology, ophthalmology, orthopaedic surgery, gastroenterology and endoscopy, anaesthesia, intensive care and urology) and from different locations (main university hospitals, ministry of health hospitals, health insurance hospitals and police hospitals). Final-year medical students were individuals who were within the last 2 years of their undergraduate medical education. That corresponds to the 6th year of study and ends with the bachelor’s exam in medicine and surgery, and to the 7th year during which they do their house officer training/internship while rotating in different departments.

### Qualitative data analysis

An iterative data analysis approach was conducted where data analysis took place concurrently with data collection [42]. The focus group discussions were audio-recorded. The data was transcribed and translated into English by the principle researcher (MS). Translated transcripts were analysed using “framework analysis involving familiarization, identifying a thematic framework, indexing, charting, mapping and interpretation” ([42], p., 178). The ATLAS.ti (a computerized indexing system, GmbH, Berlin, Germany) was employed for transcript analysis.

For construction of the coding framework, we drew upon a priori items of the push/pull factors and facilitators/barriers mentioned in the literature [17, 42]. The principle researcher (MS) identified themes and created the initial coding based on three focus group transcripts, after which data saturation was reached. AM and HP revised the coding, and a consensus process followed with work by the other researcher (MS): Finally, coding was continued with the remaining transcripts in the same manner by MS and AM. Overall, the coding was revised iteratively to reflect the data [41].

## Results

### Participants

Five focus group discussions were conducted among a total number of 18 Egyptian participants. They represent 75% of those invited. The focus group consisted of 12 residents stemming from various hospitals in different geographic locations, and 6 undergraduate medical students (2 in the sixth year and 4 in the seventh year as house officers). The focus discussions each lasted between 25 and 63 min. Demographic information of study participants is summarized in Table 1.

**Table 1:** Demographic data of study participants

Category	Residents	Students
Number	*N* = 12	*N* = 6
Gender	Male/Female = 12/0	Male/Female = 4/2
Hospital type	Primary care centres: 7 Secondary and tertiary care hospitals: 5	Tertiary care hospital: 6
Geographical location	Alexandria: 11 Tanta: 1	Alexandria: 6
Specialty	Radiology: 2 Ophthalmology: 1 Anaesthesia: 1 Orthopaedic surgery: 2 Cardiology: 3 Intensive care medicine: 1 Gastroenterology and endoscopy: 1 Urology: 1	–
Age range	26–34 years	23–26 years

### Coding framework

The coding framework was composed of two major themes and is shown in Table 2. All issues brought up by the study participants in relation to migration could be categorized within the push/pull factors and facilitator/barrier frameworks.

**Table 2:** The four themes of the coding framework

Theme	Theme title
1	Financial push and pull factors
2	Professional push and pull factors
3	Sociopolitical push and pull factors
4	Facilitators and barriers of mobility

#### Financial push and pull factors

This theme explores the financial factors driving the migration of the participating Egyptian physicians and final-year medical students. Two subthemes were identified: salary structure and the healthcare system facilities/resources. While the salary structure was perceived to be a push factor from Egypt (Table 3, Quote 1) and a pull factor for Germany (Table 3, Quote 2), financial factors were not seen as a main concern in shaping the decision for migration (Table 3, Quote 3). In comparison with other destination countries; e.g. the Gulf countries, Germany apparently has a less attractive financial power among study participants (Table 3, Quotes 4 and 5).

**Table 3:** Financial push and pull factors

	Quote	Participant
1	“Because the financial situation in Egypt is very difficult”	Male resident, location 2
2	“And I think that another benefit would be the good income which is definitely better than my income in Egypt.”	Male resident, location 7
3	“Our problem is not about finances, we don’t have any financial problems and this is not our motivation for migration”	Female house officer, location 3
4	“For me as a specialist, I can earn much more money if I worked in the Gulf countries for example; I will earn much more than I would earn in Germany but still I will take the risk because of the benefit.”	Male resident, location 6
5	“Because financial issues are not our target. If finance was my main goal, I would have travelled to Arab countries instead of Germany, but my target is to have a good life style”	Male resident, location 4
6	“There are no services available and there is a very poor infrastructure”	Male resident, location 3
7	“Lack of resources, everything… doctors, resources”	Male resident, location 5

Facilities and resources in the healthcare system were perceived as a push factor rather than a pull factor for immigration (Table 3, Quotes 6 and 7).

#### Professional push and pull factors

This theme explores the professional factors driving the migration of our study participants. Analysis of the data showed that it was perceived as one of the key factors which had a strong impact on the migration decision. However, it was more relevant for residents and house officers than it was for undergraduate students. Two subthemes were identified:

The availability of quality postgraduate training and learning opportunities in Germany was perceived as a strong attraction, and their absence created a push factor from Egypt (Table 4, Quotes 1 and 2). Physicians and students used emotional language to indicate their frustration with the lack of these opportunities in their home country; frustration and helplessness were reflected on the use of language; see the repetition 3 times of “no more” (Table 4, Quote 3).

**Table 4:** Professional push and pull factors

	Quote	Participant
1	“There is no clear system for the training even for the junior residents. They are just immersed into the new working place and expected to swim, expected to learn by doing without even respecting the guidelines.”	Male resident, location 2
2	“In Germany I will get a better training.”	Male house officer, location 1
3	“University hospitals were supposed to be the best place for the training of junior doctors; this is no longer the case. There is no more training, no more learning, no more system, everything is chaotic and disorganized.”	Male house officer, location 1
4	“Why specifically Germany? Because it has very advanced health care system and medical care; they have very advanced medical technology”	Male resident, location 1

Technology and quality of the healthcare system were mentioned as a pull factor for Germany rather than a push factor from Egypt (Table 4, Quote 4).

#### Sociopolitical push and pull factors

This theme explores the general political and sociological factors influencing the participants’ migration to Germany. Subthemes included political climate (Table 5, Quote 1), the rate of crime and violence (Table 5, Quotes 2 and 3) and improved prospects for one’s children (Table 5, Quote 4).

**Table 5:** Sociopolitical push and pull factors

	Quote	Participant
1	“I decided to leave Egypt due to all of the disappointments after the Egyptian revolution. I took the decision in 2011.”	Male resident, location 3
2	“I come from a financially stable family but I don’t like the general atmosphere or the safety level.”	Female resident, location 3
3	“I chose Germany because it is a relatively stable country, and there is freedom; this is very clear. Everyone knows that freedom is their right, unlike here.”	Male resident, location 3
4	“I am married and I have 2 daughters, I want them to live in a clean place”.	Male resident, location 5
5	I wanted to leave Egypt and work abroad and the country didn’t actually matter.	Male resident, location 2
6	“Working abroad is only a temporary, not a final situation; I plan to return back to Egypt”	Male resident, location 1
7	“Those who want to work in Germany should stay there forever and never come back. But working there and coming back after a while is useless I think”	Female resident, location 1

It is worth mentioning that the repelling power of push factors seems to have a bigger impact than the attracting power of pull factors; almost all study participants made the decision to migrate away from Egypt regardless of the choice of destination country (Table 5, Quote 5). Participants expressed mixed views regarding the migration decision; some regarded migration as a temporary decision while others made the decision to migrate permanently (Table 5, Quotes 6 and 7).

#### Facilitators and barriers of mobility

This theme explores the factors that promote or hinder the migration of participating Egyptian study participants. Five subthemes were identified: the accessibility of the German labour market, the licensing and registration procedures for foreign physicians, being a member of a social network, making use of social support resources, and signs of a culture of migration.

### German labour market

The situation in the German labour market was considered as a facilitator for migration; almost all study participants agreed on its attractiveness and easy accessibility for foreign physicians; this was mainly attributed to the shortage of physicians in Germany and the abundance of job opportunities (Table 6, Quotes 1 and 2).

**Table 6:** Facilitators and barriers to migration

	Quote	Participant
1	“I choose Germany because it is still an open labour market offering many job opportunities for doctors.”	Male resident, location 5
2	“Why specifically Germany? Because it was the only open opportunity. They need doctors.”	Male resident, location 2
3	“I think the UK is better…. The problem is the MRCP exam. I thought that the MRCP would be too difficult, it costs too much and there are so many exams to take so I chose Germany because it was easier.”	Male resident, location 5
4	“I know that the UK is much better than Germany and I knew that from the start but learning the German language was easier for me.”	Male resident, location 1
5	“I first made a trial with USLME but it was very difficult and the road was too long. So my second option was the German language.”	Male resident, location 1
6	Moderator: “You already told me that you have the first two parts of the USMLE so why didn’t you go” Participant: “Because it’s difficult to get the visa. I already applied and I booked an appointment for the clinical skills examination. But my visa was rejected twice. I applied twice but I was rejected.”	Male resident, location 5
7	“I don’t need any further exams to work as a medical doctor in Germany, there is nothing in Germany equivalent to the USMLE or MRCP. I don’t need to attend any courses for preparation. Preparation courses for USMLE or MRCP are really very difficult and time-consuming. I love the German language and it’s much easier to work in Germany than in the USA or the UK.”	Male resident, location 4
8	“Because it (Germany) is the easiest way. As a doctor all you need to learn is the German language and then you could work as a medical doctor in Germany. Other countries require accreditation of certificates and they are highly competitive.”	Male resident, location 3
9	“Why specifically Germany? It’s not specifically Germany, It’s only the fact that Germany is the easiest way out.”	Male resident, location 5
10	“Saxony is the easiest, as people say. There, there are more opportunities.”	Male resident, location 3
11	“Sure, I will start in Saxony because it is easier to get the medical license there, but I am not planning to leave as soon as I get the license. I don’t want to work in Saxony.”	Male house officer, location 1
12	“Why specifically Germany and not any other European country? Because after you have spent some time working as a medical doctor in Germany you could simply move to another European country, even to the UK. The rules have changed last year and a doctor who has been working in Germany can move to work in the UK under certain conditions. You could also migrate to Australia. So Germany gives you flexibility of moving into other countries.”	Male resident, location 5
13	“I know so many people from my study year who are already working there and I know older colleagues also.”	Male resident, location 3
14	“I depend mainly on Facebook groups as the main source for information. Doctors who are already living in Germany or who are planning to migrate create groups on Facebook to exchange knowledge and information.”	Male resident, location 2
15	“I also made some friends when I was in Germany and they helped me to find suitable accommodation and finish all the paper work needed.”	Male final year medical student, location 1
16	“I have so many friends that I helped with the application.”	Male house officer, location 1
17	“I have to read the Facebook posts about the tips and tricks regarding the required documents. The website (of the German Embassy) is so vague and unclear.”	Male resident, location 3
18	“The best thing is the experience of our colleagues. There are so many Egyptian doctors who are already working in Germany since a long time ago. So there is a big pool of experience that we can learn from.”	Male resident, location 4
19	“My uncle is a German citizen and he was always motivating me to work as a urologist just like he is. And I always wanted to be a urologist.”	Male resident, location 6
20	“People think that Germany is a paradise, and that being in Germany will automatically solve all their problems”	Female house officer, location 1
21	“I took the decision to work abroad when I was an undergraduate medical student. I talked with my colleagues, especially the older ones who have more experience…. My older colleagues … advised me to start preparing myself to work abroad.... The most important thing is to start as early as possible with the preparations for travelling. They advised us to seek any chance to leave Egypt.”	Male house officer, location 1

### Licensing and registration procedures

National licensing and registration procedures were considered to be both a facilitator and a barrier, depending on the destination country for migration. Most study participants would prefer to migrate to the USA and the UK; however they are hindered by the laborious licensing exams there, e.g. the United States Medical Licensing Examination (USMLE) or membership of the Royal College of Physicians (MRCP), respectively (Table 6, Quotes 3 and 4). Some participants had even started preparations to migrate to the USA, but were repelled by the time-consuming and expensive USMLE (Table 6, Quote 5) or the visa barrier of the USA (Table 6, Quote 6).

On the contrary, licensing and registration requirements were considered as a facilitator of migration to Germany; in many of the German federal states, a review of credentials and testing in language exams are the only assessments for an immigrating physician (Table 6, Quote 7). Germany was even described as “the easiest way out” (Table 6, Quotes 8 and 9). Within Germany, Saxony was considered one of the most preferred German federal states, in addition to its relatively easy licensing and registration requirements (Table 6, Quotes 10 and 11).

In some cases, Germany was even considered as a transit country and the “entry into Europe” rather than being the long-term destination for migration (Table 6, Quote 12).

### Social network

Analysis indicated a significant role of face-to-face social networks as well as online social network sites in facilitating migration of study participants. Both were considered to be important and reliable sources of information. In regard to inquiries about the preparation for migration, it was found useful to take advantage of connections with family, friends and colleagues who were either planning to migrate or who had already migrated. A prominent role played online interactions through Facebook groups (Table 6, Quotes 13 and 14).

### Social support

Both face-to-face social networks and online social network sites were perceived by the study participants to have a supporting function. Types of support could be classified into instrumental social support; i.e. aiding with job application procedures (Table 6, Quotes 15 and 16), informational social support, i.e. advice and exchange of important information (Table 6, Quotes 17 and 18) and emotional support in the form of care and motivation (Table 6, Quote 19).

### Culture of medical migration

Study participants expressed shared positive attitudes, beliefs and thoughts about migration. This is indicative of forming a migration culture among their relatives, colleagues and friends, and all of that facilitates and encourages further migration (Table 6, Quotes 20 and 21).

## Discussion

Migration of physicians represents a growing global phenomenon and is constantly evolving in response to the ongoing changes in the societies and heath care systems around the world. The present study – investigating a group of Egyptian physicians and final-year medical students – specifies the push and pull factors which drive their intent to migrate to Germany. Beyond the wealth gap, their particular route of migration seems chiefly determined by the requirements in licensing and registration procedures for foreign physicians in the potential destination countries. The planning and preparation for going abroad is substantially facilitated by joining a social network and community of migrating physicians with shared beliefs and practices and providing them with key information and social support. In the following we will elaborate and discuss the findings of our study.

The concept of push and pull factors has provided us with a useful framework to identify and categorize main factors influencing the decision to migrate to Germany in our group of Egyptian physicians and final year medical students. Overall, push factors to leave Egypt appeared more important than the pull factors attracting a move to Germany. This is in principle comparable to a study of South African physicians practising in Australia [4]. In our cohort, key factors for the intent to migrate are poor health care facilities, bad working conditions and poor quality of training in the source country and the conviction for better training opportunities in the destination country. This is in concordance with previous studies from South Africa, Cameroon and Pakistan [47–49]. It is however in contrast to studies from Iraq and Romania [24, 50], where the most important pull and push factors were related to salary structure and violence/terrorism. It is of notice that active recruitment activities played no obvious role in our study cohort.

The route of migration was an important theme in this qualitative study which was effected by both push and pull factors as wells as by barriers and facilitators of migration. In most cases, the decision to leave Egypt was made regardless of the choice of the destination country; the repelling power of the push factors was perceived much more strongly than the attractive power of pull factors. The destination of migration was either to the West, i.e. Europe and the USA where the professional pull factors took the upper hand, or to the East, i.e. Gulf countries where financial pull factors played the most significant role. Overall, the cohort of Egyptian physicians and final-year medical students interviewed in this study has decided to go West, thereby giving professional development factors a priority. The subsequent specific choice of the destination country is then further determined by barriers and facilitators of physician mobility. In our study, the participants apparently decided to choose Germany as the destination country itself but also due to the fact that it is part of the EU. Fundamental and facilitating reasons for this decision are the relatively low hurdles in the licensing and registrations procedures for foreign physicians by the official bodies. In some federal states of Germany, this involves merely a review of the applying foreign physician’s credentials and a test of the German language [51]. Being licensed in one German federal state automatically allows their further working as a physician anywhere else in Germany; registration by any of the other German federal states is a formal and automatic procedure. Furthermore, it may not be a surprise that some of the participating Egyptian physicians and final-years medical students see Germany primarily as a country of entry into the EU labour market and then simply plan to make use of Germany as a transition country.

In line with choosing Germany as a destination country, there are also perceived barriers associated with other potential routes for migration. While the participating Egyptian physicians and final-year medical students would have preferred the USA or the UK as an ultimate destination country, they considered the hurdle of licensing and registration procedures in those countries too high because of their requirements to pass clinical and practical medical exams (i.e. USMLE in the USA or MRCP in the UK).

As a key facilitating factor in the decision to leave Egypt and migrate to Germany emerges the formation of a local community of migrating Egyptian physicians. The course “German for doctors” provides a formal social network platform that is linked to informal social networks, such as family members, colleagues and Egyptian physicians already working abroad. The face-to-face social network is further extended by online social media network sites for migrating physicians. Facebook is regarded as a highly helpful online resource in our study cohort and actually considered to be more useful than physician’ migration-related websites which have been reported previously in the literature [17].

Our analysis indicates that these social networks serve as important sources of information transfer, identity formation and social support, e.g. for instrumental, informational and emotional assistance. These networks connect physicians and medical students planning to leave Egypt with those who have already migrated and are working successfully abroad, including those involved in a positive role-modelling. Thereby, they foster new migration in the sense of “once migration pathways are established, they will stimulate further migration” [52]. Our analysis indicates that around the theme “migration of Egyptian physicians to Germany” a community has developed with its own culture, where the community members offer shared understandings and beliefs of their current situation, including a positive attitude towards migration. They also show shared practices in their planning and preparation procedures. The community transfers the knowledge needed between their members, including the transfer from generations of physicians – who have already successfully migrated – to the future generations of physicians still intending to migrate [53].

Beyond the study itself, our findings may yield a few general implications and perspectives. First, Germany is part of an international carousel for migrating physicians, i.e. it is a destination and a source country at the same time. Leading destination countries are Switzerland, Austria, United Kingdom and the USA [36]. Overall, it seems true that Germany “loses more doctors to emigration than it gains by immigration” and this results in a relative shortage of physicians. ([36], p. 37). Secondly, the EU may consider establishing a general framework for the licensing and registration requirement for non-EU physicians entering, similar to the framework already undertaken to manage the migration of physicians within the EU. Low hurdle procedures in one or more EU countries may potentially impair the quality of the healthcare system and patients’ safety in those countries, but due to the free mobility of physicians within the EU, it could still affect the patients’ care in the other EU countries.

This qualitative research study has some limitations. One is language, because the focus groups’ discussions were conducted in the participants’ native language, namely Arabic. The translation into English may have affected the original meaning that is constructed rather than expressed by language [54]. Translation also compromises a full discourse analysis and was a barrier against listening to audiotape while reading transcripts; that is a step which would have ensured more accuracy of interpretation [42]. Another limitation is selection bias. We invited only physicians and final-year medical students who are attending the preparatory course for the medical language examination in Germany. Most of our study participants were male physicians; although, this represents the male-to-female ratio of the “German for doctors” course participants. This may be a source of bias but it reflects the conservative Egyptian culture where most families don’t allow their daughters to travel long distances even within Egypt, or let them alone migrate to Europe [55]. Furthermore, the findings of this study represent the experiences and views of Egyptian physicians and final-year medical students. That should not be generalized to physicians migrating in from other countries.

## Conclusions

The migration of Egyptian physicians and final-year medical students to Germany is driven by a specific weighting of push and pull factors. Push factors are more important than pull factors, and professional development factors play a leading role. The route of migration is mainly determined by the importance of low hurdle registration and licensing requirements in the destination country. The planning and preparation of migration is substantially facilitated by the local formation of a community and culture of migration with multiple sources for information exchange, identify formation and social support through face-to-face and online channels.
